# Equipping the 8th Edition American Joint Committee on Cancer Staging for Gastric Cancer with the 15-Node Minimum: a Population-Based Study Using Recursive Partitioning Analysis

**DOI:** 10.1007/s11605-017-3504-0

**Published:** 2017-07-27

**Authors:** Shu-Qiang Yuan, Yu-Tong Chen, Ze-Ping Huang

**Affiliations:** 1 Department of Gastric Surgery, Sun Yat-sen University Cancer Center, State Key Laboratory of Oncology in South China, Collaborative Innovation Center for Cancer Medicine, 651 Dong Feng Road East, Guangzhou, 510060 China; 2 Department of Medical Oncology, Sun Yat-sen University Cancer Center, State Key Laboratory of Oncology in South China, Collaborative Innovation Center for Cancer Medicine, Guangzhou, 510060 China; 3 Department of General Surgery, Lan Zhou University Second Hospital, Lanzhou, 730030 China

**Keywords:** Gastric cancer, American Joint Committee on Cancer staging, Evaluated lymph node, Recursive partitioning analysis, Surveillance, Epidemiology, and End Results

## Abstract

**Bakcground:**

The recently proposed 8th American Joint Committee on Cancer (AJCC) staging for gastric cancer (GC) did not include the evaluated lymph node (ELN) count as a prognostic indicator. In this study, we performed recursive partitioning analysis (RPA) to objectively combine the 15-ELN threshold and 8th AJCC stage to refine the staging for GC.

**Methods:**

We analyzed 19,018 patients with non-metastatic GC from the Surveillance, Epidemiology, and End Results database. The dataset was randomly divided into training and validation sets.

**Results:**

For each 8th AJCC stage, survival was significantly better for patients with ≥15 ELNs versus those with <15 ELNs (*P* < 0.001 for all). RPA divided non-metastatic GC into seven stages: RPA-IA (8th AJCC IA with ≥15 ELNs), RPA-IB (IA with <15 ELNs and IB/IIA with ≥15 ELNs), RPA-IIA (IB with <15 ELNs and IIB with ≥15 ELNs), RPA-IIB (IIA with <15 ELNs and IIIA with ≥15 ELNs), RPA-IIIA (IIB with <15 ELNs), RPA-IIIB (IIIA with <15 ELNs and IIIB ≥15 ELNs), and RPA-IIIC (IIIB with <15 ELNs and IIIC). The corresponding 5-year survival rates were 84.1, 70.3, 52.8, 41.4, 32.9, 21.7, and 10.2%, respectively (*P* < 0.001 for all pairwise comparisons). The RPA staging outperformed the 8th AJCC staging in terms of discrimination and homogeneity among the SEER training and validation sets, as well as an independent Chinese cohort.

**Conclusion:**

By equipping the 8th AJCC stage with the 15-ELN threshold, the proposed RPA staging is superior to the 8th AJCC staging without overcomplicating.

**Electronic supplementary material:**

The online version of this article (doi:10.1007/s11605-017-3504-0) contains supplementary material, which is available to authorized users.

## Introduction

For patients with gastric cancer (GC), accurate survival prediction is pivotal to treatment planning and surveillance. Currently, the American Joint Committee on Cancer (AJCC) TNM classification is the most commonly used prognostic system for patients with GC.^1^ The 7th edition AJCC staging scheme for GC, which was based on Japanese and Korean databases and published in 2010,^1^ has been evaluated by a number of studies.^2^–^5^ Although most of these studies confirmed its prognostic value, the 7th AJCC N classification is merely based on the positive lymph node count and has been criticized for disregarding the impact of the evaluated lymph node (ELN) count on survival.^6^–^8^ Moreover, although the 7th AJCC staging scheme recognized the prognostic value of N3b, it did not incorporate N3b into the stage grouping.

The 8th edition AJCC staging scheme for GC has been launched recently.^9^ It was based on a multi-institutional cohort collected by the International Gastric Cancer Association with a large sample size (>25,000 cases) and abundant geographic variety.^10^ In the 8th AJCC staging scheme, N3a and N3b were designated as separate groups in the stage grouping, that is, T4aN3a, T1N3b, T2N3b, and T3N3b, which were previously classified into IIIC, IIB, IIIA, and IIIB, respectively, in the 7th AJCC staging and were re-classified into IIIB, IIIB, IIIB, and IIIC, respectively, in the 8th edition.^11^ Moreover, the 8th AJCC staging scheme exhibited improved discriminatory ability as compared with the 7th edition, especially in stage III.^11^

A minimum of 16 ELNs is necessary to identify N3b disease, and the National Comprehensive Cancer Network guidelines for GC recommend harvesting ≥15 ELNs for accurate staging.^12^ But in general clinical practice, the ELN count differs according to various factors, and the compliance to the 15-ELN threshold is generally poor in the USA.^6^ Nonetheless, the 8th AJCC staging scheme for GC did not include the ELN count as a prognostic indicator. We hypothesized that equipping the 8th AJCC staging system with the 15-ELN threshold would further improve its prognostic accuracy.

In the present study, we developed a novel staging scheme for non-metastatic GC by using the recursive partitioning analysis (RPA),^13^,^14^ which can achieve the optimized combination of the 15-ELN threshold and the 8th AJCC stage. The aim of this study is to improve the prognostic performance of the 8th AJCC staging without overcomplicating.

## Patients and Methods

### Study Cohort

From the National Cancer Institute’s Surveillance, Epidemiology, and End Results (SEER) database (18 SEER registries), we identified 89,367 aged 18 and older patients with GC (NAACCR item no. 400, codes C16.0–C16.9) from January 2000 to December 2013. We limited the time period after the year of 2000 in order to include the cases from all the 18 SEER registries. Patients without histologic diagnosis, with a history of prior or concurrent malignancies, carcinoma in situ, distant metastasis, and with missing information regarding T stage, the positive lymph node count or the ELN count were excluded. The final analytic cohort consisted of 19,018 patients with non-metastatic GC. All patients were restaged by the 8th AJCC staging scheme.

An independent Chinese cohort of patients (1446 cases) who had undergone radical gastrectomy and D2 lymphadenectomy for GC between 2001 and 2010 in the Sun Yat-Sen University Cancer Center was used as validation data. The Chinese cohort was collected according to the same inclusion and exclusion criteria. The study protocol for the Chinese cohort was approved by the independent Ethics Committee of Sun Yat-Sen University Cancer Center.

### Statistical Analysis

The change in the proportion of patients with ≥15 ELNs in the SEER cohort during 2000–2013 was assessed using the Cochran-Armitage test for trend. Only patients with at least 3-year follow-up (2000–2010, 15,466 cases) were included in survival analyses. The Kaplan-Meier method and the log-rank test were used to compare overall survival (OS) between patients with ≥15 ELNs and <15 ELNs within each of the 8th AJCC stages.

Two thirds of the patients with at least 3-year follow-up in the SEER cohort were randomly assigned to a training set (10,319 cases) and the remaining one third were assigned to a validation set (5147 cases) to develop and validate a more powerful staging scheme which combined the prognostic information of 8th AJCC staging and the 15-ELN threshold. The recursive partitioning analysis (RPA) is based on the optimized binary partition of these subgroups which results in new subgroups with relatively homogeneous prognosis and maximum survival discrimination between these subgroups.^13^,^14^ We performed RPA to generate a novel RPA staging scheme by regrouping the following seven pairs of patient subgroups: 8th AJCC IA with ≥15 and <15 ELNs, IB with ≥15 and <15 ELNs, IIA with ≥15 and <15 ELNs, IIB with ≥15 and <15 ELNs, IIIA with ≥15 and <15 ELNs, IIIB with ≥15 and <15 ELNs, and IIIC with ≥15 and <15 ELNs. Multivariate Cox proportional hazards regression was used to examine the association between the RPA stage and hazard ratio (HR) for death after adjustment for clinicopathologic factors.

In the training set, the SEER validation set, and the Chinese cohort, the comparative performances of the RPA staging and the 8th AJCC staging schemes were assessed in terms of discriminatory ability and prognostic homogeneity. The discriminatory capacity of the staging schemes was measured using the concordance index (C-index)^15^ and the Akaike’s information criterion (AIC). The higher the C-index or the lower the AIC value, the greater the discrimination of the staging scheme. Likelihood ratio *χ* ^2^ tests related to the Cox regression models were used to measure the prognostic homogeneity of the staging schemes. The greater the Likelihood ratio *χ* ^2^ value, the better the prognostic homogeneity of the staging scheme.

Statistical significance was set as *P* < 0.050 in a two-tailed test. The statistical analyses were performed using IBM SPSS Statistics for Windows v.19.0 (IBM Corp., Armonk, NY, USA) and R v. 3.3.1 (http://www.r-project.org).

## Results

Table 1 summarizes the demographic and cancerous characteristics of the SEER cohort (19,018 cases). The majority of the patients had node-positive disease (60.6%) and <15 ELNs (54.1%). The mean positive lymph node and ELN counts were 4.3 ± 6.7 and 16.1 ± 12.0, respectively. The 5-year OS rate for patients in the study cohort was 39.3%. The 1446 patients in the Chinese cohort had clinicopathologic features distinct from those in the SEER cohort, particularly in terms of the percentage of patients with ≥15 ELNs (69.1%; Supplementary Table 1).

**Table 1:** Clinicopathologic features of the study cohort

Variable	*N* (%)
Age (years)	
<50	2355 (12.4)
50–59	3280 (17.2)
60–69	4609 (24.2)
70–79	5434 (28.6)
≥80	3340 (17.6)
Sex	
Male	11,434 (60.1)
Female	7584 (39.9)
Race	
Non-Hispanic white	8873 (46.7)
Non-Hispanic black	2533 (13.3)
Hispanic	3444 (18.1)
Other	4168 (21.9)
Marital status	
Married	11,585 (60.9)
Widowed	2910 (15.3)
Other	4523 (23.8)
Year of diagnosis	
2000–2003	5991 (31.5)
2004–2007	5564 (29.3)
2008–2013	7463 (39.2)
SEER region	
Midwest	2017 (10.6)
Northeast	3413 (17.9)
South	3148 (16.6)
West	10,440 (54.9)
Tumor location	
Upper one third	4668 (24.5)
Middle one third	1711 (9.0)
Lower one third	6142 (32.3)
Other/unspecified	6497 (34.2)
Tumor size (cm)	
<2	2224 (11.7)
2–3.9	4689 (24.7)
4–5.9	4301 (22.6)
≥6	5401 (28.4)
Unknown	2403 (12.6)
Tumor grade	
G1/G2	5723 (30.1)
G3/G4	12,419 (65.3)
Unknown	876 (4.6)
T stage	
T1	4145 (21.8)
T2	2333 (12.3)
T3	6996 (36.8)
T4a	3881 (20.4)
T4b	1663 (8.7)
Mean positive node count (SD)	4.3 (6.7)
N stage	
N0	7497 (39.4)
N1	3501 (18.4)
N2	3532 (18.6)
N3a	3233 (17.0)
N3b	1255 (6.6)
Mean ELN count (SD)	16.1 (12.0)
The 15-node threshold	
<15	10,285 (54.1)
≥15	8733 (45.9)

*SEER* Surveillance, Epidemiology, and End Results, *PLN* positive lymph node, *ELN* evaluated lymph node

**Fig. 1:**
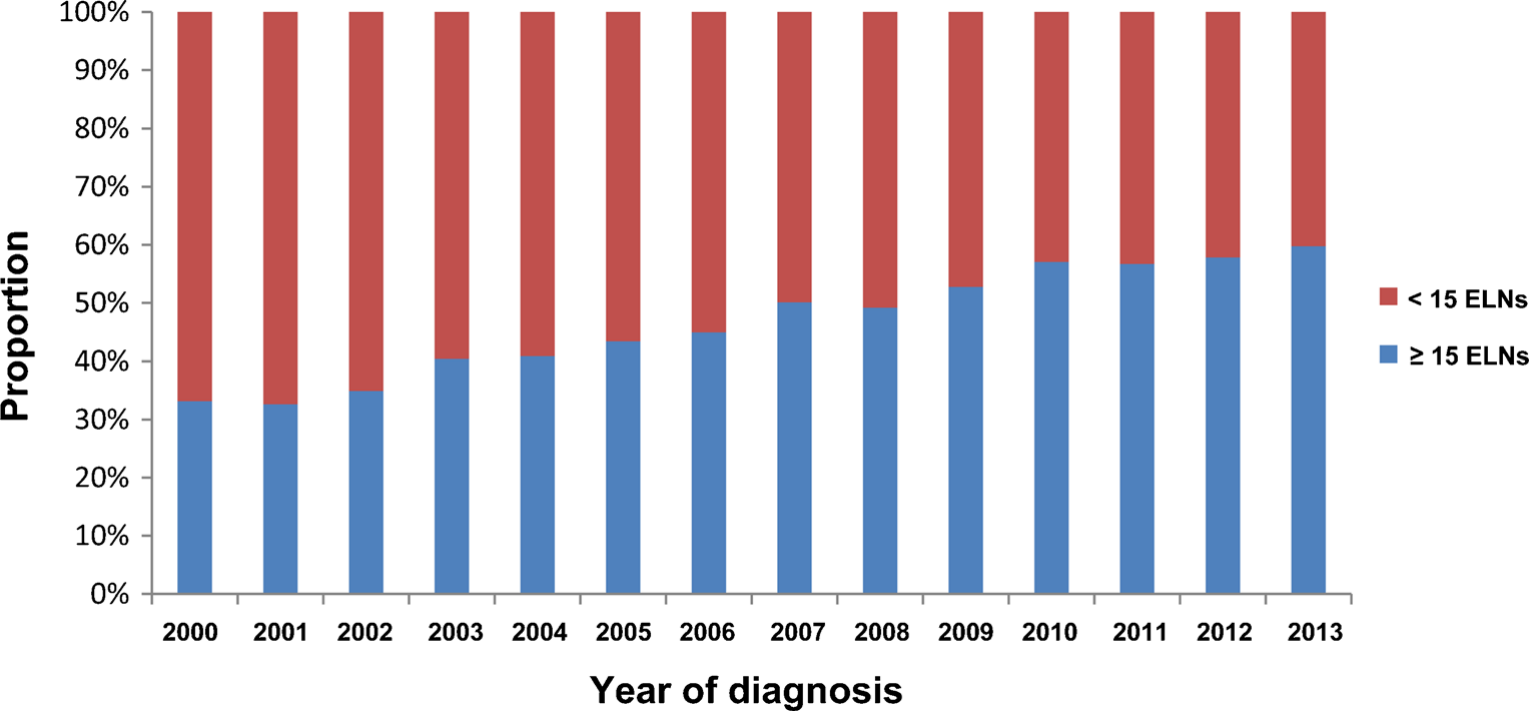
The change in the proportion of patients with ≥15 lymph nodes evaluated over the period of 2000–2013. *ELN* evaluated lymph node

As shown in Fig. 1, the proportion of patients with ≥15 ELNs increased significantly from 33.2% in 2000 to 59.7% in 2013 (*P*_trend_ < 0.001). For each of the 8th AJCC stages, survival was significantly better for patients with ≥15 ELNs compared with those with <15 ELNs (*P* < 0.001 for all; Table 2). Of note, patients within the 8th stage IIA (5-year OS rate, 48.9%) was further stratified by the 15-ELN threshold into subgroups with remarkably different prognosis, and an almost 20% difference in the 5-year OS rates was identified between patients with <15 ELNs and those with ≥15 ELNs (41.9 vs. 61.7%, *P* < 0.001; Table 2). In the Chinese cohort treated with D2 lymphadenectomy, the 15-ELN threshold was also a significant prognostic factor independent of AJCC stage and other clinicopathologic factors (HR for ≥15 vs. <15 ELNs, 0.52 [95% CI, 0.43–0.62]; *P* < 0.001).

**Table 2:** The 5-year overall survival (OS) stratified by the evaluated lymph node (ELN) count within each 8th American Joint Committee on Cancer (AJCC) stage

8th AJCC stage	5-year OS rate			HR^a^ (95% CI)	*P* value^a^
	All (%)	<15 ELNs (%)	≥15 ELNs (%)		
IA	73.6	71.0	79.1	0.77 (0.66–0.89)	<0.001
IB	58.8	53.4	70.1	0.79 (0.67–0.93)	0.004
IIA	48.9	41.9	61.7	0.64 (0.56–0.72)	<0.001
IIB	34.5	28.4	45.6	0.63 (0.56–0.70)	<0.001
IIIA	24.5	18.9	32.7	0.64 (0.59–0.69)	<0.001
IIIB	16.1	11.4	21.7	0.65 (0.60–0.71)	<0.001
IIIC	8.8	6.8	10.5	0.83 (0.70–0.99)	0.036

*HR* hazard ratio, *CI* confidence interval

^a^Adjusted for age, sex, race, year of diagnosis, marital status, SEER region, tumor site, tumor diameter, and tumor grade

**Fig. 2:**
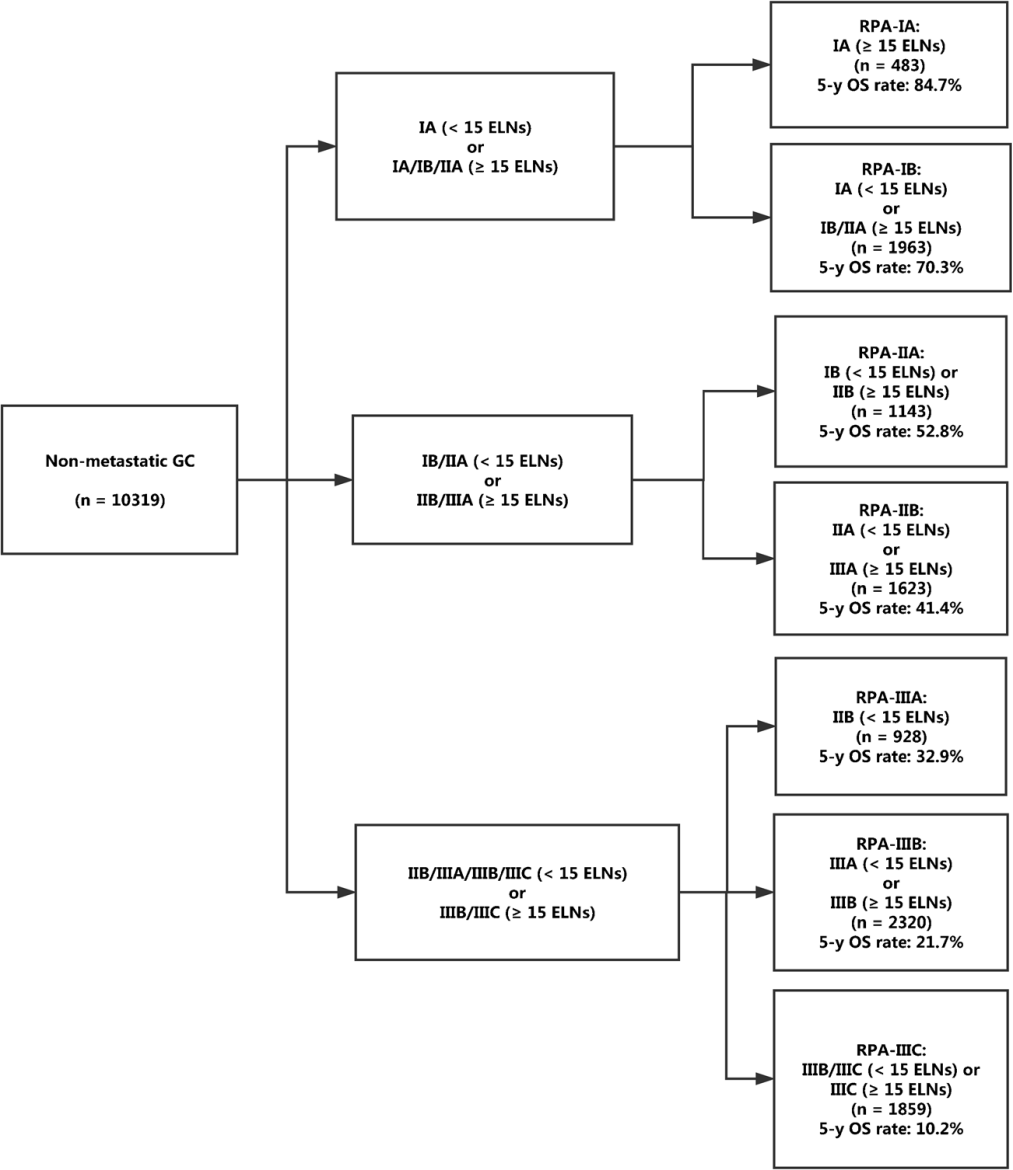
The process of stage regrouping for non-metastatic gastric cancer on the basis of recursive partitioning analysis. *GC* gastric cancer, *ELN* evaluated lymph node, *RPA* recursive partitioning analysis, *OS* overall survival

The demographic and cancerous characteristics were comparable among the training set (10,319 cases) and the SEER validation set (5147 cases) (Supplementary Table 2). On the basis of RPA, patients in the training set were classified into the following seven novel stage groups (Fig. 2): RPA-IA (8th AJCC IA with ≥15 ELNs), RPA-IB (AJCC IA with <15 ELNs and IB/IIA with ≥15 ELNs), RPA-IIA (AJCC IB with <15 ELNs and IIB with ≥15 ELNs), RPA-IIB (AJCC IIA with <15 ELNs and IIIA with ≥15 ELNs), RPA-IIIA (AJCC IIB with <15 ELNs), RPA-IIIB (AJCC IIIA with <15 ELNs and IIIB ≥15 ELNs), and RPA-IIIC (AJCC IIIB with <15 ELNs and IIIC).

**Fig. 3:**
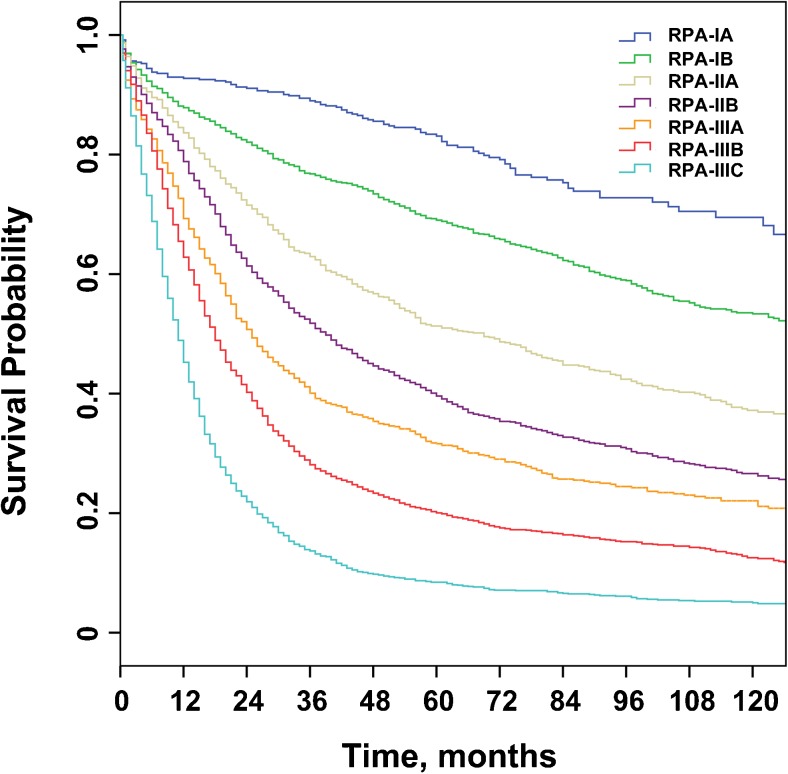
Overall survival of patients with non-metastatic gastric cancer stratified by the proposed staging scheme. *RPA* recursive partitioning analysis

For the training set, there were 483 (4.7%), 1963 (19.0%), 1143 (11.1%), 1623 (15.7%), 928 (9.0%), 2320 (22.5%), and 1859 (18.0%) patients in the RPA-IA, IB, IIA, IIB, IIIA, IIIB, and IIIC stage groups, respectively. The corresponding 5-year OS rates were 84.1, 70.3, 52.8, 41.4, 32.9, 21.7, and 10.2%, respectively (*P* < 0.001 for all pairwise comparisons; Fig. 3). After adjusted for age, sex, race, year of diagnosis, marital status, SEER region, tumor site, tumor diameter, and tumor grade, we confirmed that a higher RPA stage was associated with an increased hazard of mortality (RPA-IB vs. RPA-IA: HR, 1.56; RPA-IIA vs. RPA-IA: HR, 2.40; RPA-IIB vs. RPA-IA: HR, 3.26; RPA-IIIA vs. RPA-IA: HR, 4.07; RPA-IIIB vs. RPA-IA: HR, 5.74; RPA-IIIC vs. RPA-IA: HR, 9.45; *P* < 0.001 for all).

As shown in Table 3, patients within the 8th AJCC stages IA–IIIB can be further stratified by the RPA staging into subgroups with remarkably different 5-year OS rates (absolute differences in the 5-year OS rates ≥10% and *P* < 0.001 for all 8th AJCC stages). For instance, patients with 8th stage IIB disease (5-year OS rate, 34.5%) could be further stratified into RPA-IB and RPA-IIB subgroups, and a 20.4% difference in the 5-year OS rates was found between patients classified as having RPA-IB and those classified as having RPA-IIB disease (47.7 vs. 27.3%, *P* < 0.001).

**Table 3:** The 5-year overall survival (OS) within each 8th American Joint Committee on Cancer (AJCC) stage stratified by the RPA stage

8th AJCC stage	5-year OS rate							HR (95% CI)^a,b^	*P* value^a^
	RPA-IA (%)	RPA-IB (%)	RPA-IIA (%)	RPA-IIB (%)	RPA-IIIIA (%)	RPA-IIIB (%)	RPA-IIIIC (%)		
IA	79.8	69.3	–	–	–	–	–	1.26 (1.08–1.48)	<0.001
IB	–	67.0	53.0	–	–	–	–	1.29 (1.09–1.53)	0.004
IIA	–	62.6		41.8	–	–	–	1.28 (1.20–1.37)	<0.001
IIB	–	–	47.7		27.3	–	–	1.24 (1.17–1.31)	<0.001
IIIA	–	–	–	32.4	–	19.2	–	1.26 (1.21–1.31)	<0.001
IIIB	–	–	–	–	–	18.5	8.5	1.61 (1.49–1.75)	<0.001
IIIC							9.5	Not applicable^c^	Not applicable^c^

*RPA* recursive partitioning analysis, *HR* hazard ratio, *CI* confidence interval

^a^Adjusted for age, sex, race, year of diagnosis, marital status, SEER region, tumor site, tumor diameter, and tumor grade

^b^The lower RPA stage as the comparison group

^c^Because the patients within the 8th AJCC stage IIIC were all classified into RPA-IIIC, stratified survival analysis was not performed

The RPA staging scheme achieved a C-index of 0.681 (95% CI, 0.674–0.688), 0.687 (95% CI, 0.677–0.697), and 0.720 (95% CI, 0.695–0.745) in the training set, the SEER validation set, and the Chinese set, respectively, which was significantly superior to the 8th AJCC staging system (training set: C-index, 0.665; 95% CI, 0.658–0.672; *P* < 0.001; SEER validation set: C-index, 0.674; 95% CI, 0.664–0.684; *P* = 0.008; Chinese set: C-index, 0.702; 95% CI, 0.677–0.947; *P* = 0.036). Moreover, among the training set and the two validation sets, the RPA staging scheme outperformed the 8th AJCC staging scheme in terms of the AIC (for the training set, 119,107.7 vs. 119,433.6; for the SEER validation set, 54,274.6 vs. 54,411.3; for the Chinese set, 6877.5 vs. 6929.2) and in the likelihood ratio *χ* ^2^ test (likelihood ratio *χ* ^2^ value 2574.5 vs. 2258.21 in the training set, 1402.9 vs. 1242.7 in the SEER validation set, and 326.2 vs. 274.5 in the Chinese set).

## Discussion

In this study of patients with non-metastatic GC from the SEER database, we demonstrate a significantly better survival for patients with ≥15 ELNs compared with those with <15 ELNs within each 8th AJCC stage. Thus, we performed RPA to develop a novel staging scheme for non-metastatic GC which incorporated the prognostic information of the 15-ELN threshold and 8th AJCC stage.

In the training set, we demonstrated significant prognostic heterogeneity within six of the seven 8th AJCC stages (from IA to IIIB) when stratified by the RPA stage. For instance, the 8th stage IIB disease was further stratified into RPA-IB and RPA-IIA disease, and the difference in the 5-year OS rates between patients in these two RPA stages exceeded 20%. Furthermore, even 8th AJCC stages IA and IIIB, which were at the extremes of the 8th AJCC staging, could be further stratified into RPA stages with a ≥ 10% difference in the 5-year OS rates. Moreover, in the training set, the SEER validation set, and the Chinese set, the RPA staging scheme outperformed the 8th AJCC staging scheme in terms of all the parameters measuring discriminatory ability and prognostic homogeneity, which suggests minimal evidence of model overfit and the potential generalizability of the proposed RPA staging scheme.

As shown in this US population-based study, although the compliance with the 15-ELN threshold has improved over the past decade, it is still unsatisfactory (59.7%) even in the year of 2013. The most possible reason is that the more extensive lymphadenectomy is generally poorly accepted in the USA^6^,^16^ because several randomized control trials have failed to demonstrate significant OS benefits for such invasive surgery.^17^–^20^ Additionally, as the ELN count might differ according to individual physical condition, operation condition, and pathological examination,^21^ it is hard to ensure harvesting of ≥15 ELNs in each patient in routine clinical practice. In the recently proposed 8th edition AJCC staging scheme, N3b (>15 positive nodes) was incorporated in the stage grouping. Since >15 ELNs are required to identify N3b disease, the prognostic information of N3b is unavailable in a large proportion of the US population with GC. Thus, the proposed RPA staging scheme, which equipped the 8th AJCC staging with the 15-ELN threshold, is of great value in routine clinical practice in the USA.

A number of prognostic nomograms, which combined the prognostic information of various prognostic factors, have been proposed to improve the prognostic accuracy among patients with GC.^22^–^25^ However, these nomograms have not been popularized so far, probably because they are inherently complex and inconvenient to apply. In contrast, although the proposed RPA staging scheme incorporated the 15-ELN threshold and the 8th AJCC stage, it is still a simple system consisting of seven well-defined stage groups. Thus, it is noteworthy that the improved prognostic power of the proposed RPA staging scheme compared with the 8th AJCC staging scheme was not at the cost of overcomplicating and that the proposed RPA staging scheme is ease of use in treatment planning and surveillance.

The underling mechanisms for the prognostic impact of the ELN count remain unclear. One possible explanation is that patients with an inadequate ELN count might be understaged; the increase in the ELN count may improve the prognostic accuracy for patients with resected GC and thus lead to more appropriate postoperative treatments and improved survival.^26^ Additionally, the number of ELNs may represent a surrogate for the quality GC surgery.^21^ Therefore, removing a greater number of lymph nodes might lower the risk of residual positive nodes and nodal micrometastases and may thus lower the risk of recurrence. However, since the ELN count was dependent on both the number of nodes removed by surgeons and those examined by pathologists, we were not able to separate the therapeutic effect of lymph node dissection from the stage migration effect. Moreover, it was speculated that in patients with a strong immune response, the resulting enlargement of lymph nodes may make them easier to recognize and retrieve, leading to the observed better survival in patients with a higher ELN count.^27^

We acknowledge that the present study has several limitations. First, although the SEER database makes great efforts to ensure the accuracy and quality of data, miscoding could still exist. Second, information on patient comorbidities and performance status, extent of lymphadenectomy, and chemotherapy is not available in the SEER database. Since OS is the primary endpoint in this study, medical comorbidities or other competing causes of death might influence our results. However, OS is the most valuable endpoint for cancer patients and has a unified definition across different hospitals. Additionally, because the extent of lymphadenectomy was unavailable, we could not draw solid conclusions on the therapeutic effect of the extended lymphadenectomy. Moreover, because information regarding chemotherapy was not available in the SEER database, future studies are needed to assess how the proposed RPA staging may influence decision-making regarding postoperative therapies. Third, external validation using patient cohorts from other countries outside the USA and China is required.

In summary, we demonstrate that harvesting ≥15 ELNs was associated with a better survival across all 8th AJCC stages for non-metastatic GC, which suggests that the prognostic accuracy of the 8th AJCC staging needs improvement. Accordingly, we derived a novel RPA staging scheme which incorporated the prognostic information of the 15-ELN threshold and 8th AJCC stage. The RPA staging outperformed the 8th AJCC staging without overcomplicating. The proposed RPA staging system will be clinically useful for prognosis and decision-making regarding treatment and surveillance among patients with non-metastatic GC.

AIC, Akaike’s information criterion; AJCC, American Joint Committee on Cancer; C-index, concordance index; ELN, evaluated lymph node; GC, gastric cancer; HR, hazard ratio; RPA, recursive partitioning analysis; SEER, Surveillance, Epidemiology, and End Results

## Electronic Supplementary Material

Supplementary Table 1 (DOC 48 kb)

Supplementary Table 2 (DOC 72 kb)
