# Supplementary material for: Equipping the 8th Edition American Joint Committee on Cancer Staging for Gastric Cancer with the 15-Node Minimum: a Population-Based Study Using Recursive Partitioning Analysis
Source: J Gastrointest Surg. 2017 Jul 27;21(10):1591–8. doi: 10.1007/s11605-017-3504-0 (PMC5610217; doi:10.1007/s11605-017-3504-0)
Supplement: Supplementary file 1 — (DOC 48 kb) [file 11605_2017_3504_MOESM1_ESM.doc]

**Supplementary Table 1.** Clinicopathologic features of the Chinese cohort

| **Variable** | ***N* (%)** |
| --- | --- |
| **Mean age, years (SD)** | 57.8 (11.6) |
| **Sex** |  |
| Male | 967 (66.9) |
| Female | 479 (33.1) |
| **Tumor location** |  |
| Upper one third | 500 (34.6) |
| Middle one third | 238 (16.5) |
| Lower one third | 708 (49.0) |
| **Mean tumor size, cm (range)** | 4.6 (2.9) |
| **Tumor grade** |  |
| G1/G2 | 425 (29.4) |
| G3/G4 | 1021 (70.6) |
| **T stage** |  |
| T1 | 146 (10.1) |
| T2 | 192 (13.3) |
| T3 | 306 (21.2) |
| T4a | 667 (46.1) |
| T4b | 135 (9.3) |
| **Mean positive node count (SD)** | 5.1 (7.0) |
| **N stage** |  |
| N0 | 485 (33.5) |
| N1 | 248 (17.2) |
| N2 | 303 (21.0) |
| N3a | 288 (19.9) |
| N3b | 122 (8.4) |
| **Mean ELN count (SD)** | 21.7 (11.9) |
| **The 15-node threshold** |  |
| < 15 | 447 (30.9) |
| ≥ 15 | 999 (69.1) |

PLN, positive lymph node; ELN, evaluated lymph node.
