# Supplementary material for: Equipping the 8th Edition American Joint Committee on Cancer Staging for Gastric Cancer with the 15-Node Minimum: a Population-Based Study Using Recursive Partitioning Analysis
Source: J Gastrointest Surg. 2017 Jul 27;21(10):1591–8. doi: 10.1007/s11605-017-3504-0 (PMC5610217; doi:10.1007/s11605-017-3504-0)
Supplement: Supplementary file 2 — (DOC 72 kb) [file 11605_2017_3504_MOESM2_ESM.doc]

**Supplementary Table 2.** Clinicopathologic characteristics of the training set and the validation set

| **Variable** | ***N* (%)** | |
| --- | --- | --- |
|  | **Training set**  **(n = 10319)** | **Validation set**  **(n = 5147)** |
| **Age, years** |  |  |
| < 50 | 1304 (12.6) | 2355 (12.4) |
| 50−59 | 1727 (16.7) | 3280 (17.2) |
| 60−69 | 2455 (23.8) | 4609 (24.2) |
| 70−79 | 2972 (28.8) | 5434 (28.6) |
| ≥ 80 | 1861 (18.0) | 3340 (17.6) |
| **Sex** |  |  |
| Male | 6236 (60.4) | 3069 (59.6) |
| Female | 4083 (39.6) | 2078 (40.4) |
| **Race** |  |  |
| Non-Hispanic white | 4959 (48.1) | 2500 (48.6) |
| Non-Hispanic black | 1352 (13.1) | 690 (13.4) |
| Hispanic | 1785 (13.7) | 891 (17.3) |
| Other | 2223 (21.5) | 1066 (20.7) |
| **Marital status** |  |  |
| Married | 6357 (61.6) | 3102 (60.3) |
| Widowed | 1671 (16.2) | 796 (15.5) |
| Other | 2291 (22.2) | 1249 (24.3) |
| **Year of diagnosis** |  |  |
| 2000−2003 | 3975 (38.5) | 2016 (39.2) |
| 2004−2007 | 3711 (36.0) | 1853 (36.0) |
| 2008−2013 | 2633 (25.5) | 1278 (24.8) |
| **SEER region** |  |  |
| Midwest | 1117 (10.8) | 550 (10.7) |
| Northeast | 1899 (18.4) | 957 (18.6) |
| South | 1679 (16.3) | 885 (17.2) |
| West | 5624 (54.5) | 2755 (53.5) |
| **Tumor location** |  |  |
| Upper one third | 2659 (25.8) | 1266 (24.6) |
| Middle one third | 885 (8.6) | 441 (8.6) |
| Lower one third | 3268 (31.7) | 1673 (32.5) |
| Other/unspecified | 3507 (34.0) | 1767 (34.3) |
| **Tumor size, cm** |  |  |
| < 2 | 1132 (11.0) | 541 (10.5) |
| 2−3.9 | 2462 (23.9) | 1250 (24.3) |
| 4−5.9 | 2372 (23.0) | 1161(22.6) |
| ≥ 6 | 2965 (28.7) | 1511 (29.4) |
| Unknown | 1388 (13.5) | 684 (13.3) |
| **Tumor grade** |  |  |
| G1/G2 | 3039 (29.5) | 1564 (30.4) |
| G3/G4 | 6779 (65.7) | 3344 (65.0) |
| Unknown | 501 (4.9) | 239 (4.6) |
| **T stage** |  |  |
| T1 | 2120 (20.5) | 1066 (20.7) |
| T2 | 1260 (12.2) | 633 (12.3) |
| T3 | 3873 (37.5) | 1976 (38.4) |
| T4a | 2087 (20.2) | 999 (19.4) |
| T4b | 979 (9.5) | 473 (9.2) |
| **Mean positive node count (SD)** | 4.4 (6.8) | 4.2 (6.3) |
| **N stage** |  |  |
| N0 | 3922 (38.0) | 1989 (38.6) |
| N1 | 1930 (18.7) | 969 (18.8) |
| N2 | 1989 (19.3) | 988 (19.2) |
| N3a | 1779 (17.2) | 884 (17.2) |
| N3b | 699 (6.8) | 317 (6.2) |
| **Mean ELN count (SD)** | 15.3 (11.6) | 15.2 (11.7) |
| **The 15-node threshold** |  |  |
| < 15 | 5854 (56.7) | 2941 (57.1) |
| ≥ 15 | 4465 (43.3) | 2206 (42.9) |

SEER, Surveillance, Epidemiology, and End Results; ELN, evaluated lymph node.
